# Supplementary material for: CD8 and CD4 T Cell Populations in Human Kidneys
Source: Cells. 2021 Feb 1;10(2):288. doi: 10.3390/cells10020288 (PMC7912772; doi:10.3390/cells10020288)
Supplement: Supplementary file 1 [file cells-10-00288-s001.pdf]

Table of contents: Supplemental Materials

	Title	relates to main figures
**Table S1**	Monoclonal antibodies used for phenotyping.	1–5
**Table S2**	Monoclonal antibodies used for stimulation assay.	6
**Figure S1**	Gating strategy T cell phenotyping.	1–5
**Figure S2**	Gating strategy polyfunctional assay.	6
**Figure S3**	CD3 cell counts.	1
**Figure S4**	Phenotype of CD8 and CD4 T cells derived from PB and healthy kidney.	1 and 2
**Figure S5**	CD27/CD45RA/CCR7/CD28-expression profile and phenotype of CD8 and CD4 T cells from healthy kidney compared to TX kidney.	3
**Figure S6**	Phenotype of CD69/CD103 based subsets in CD8 and CD4 T cells derived from kidney.	4
**Figure S7**	Cytokines produced by healthy kidney compared to TX kidney derived CD8 and CD4 T cells.	7

Abbreviations and acronyms

BKPyV	polyomavirus BK
ADPKD	autosomal dominant polycystic kidney disease
ATN	acute tubular necrosis
CMV	cytomegalovirus
FSGS	focal segmental glomerulosclerosis
GM-CSF	granulocyte-macrophage colony stimulating factor
HB	heartbeating
Hobit	homolog of Blimp1 in T cells
IFNγ	interferon-γ
IgAN	nephropathy
IL-17A	interleukin-17
IL-2	interleukin-2
IL-7Rα	α-chain of interleukin receptor 7
KLF2	Krüppel-like Factor 2
KLRG1	Killer cell lectin-like receptor subfamily G member 1
LTAG	large T antigen
MAIT	mucosa-associated invariant T cells
MNC	Mononuclear cells
NHB	non-heartbeating
PB	Peripheral blood
RTRs	renal transplant recipients
S1PR1	Sphingosine-1-phosphate receptor 1
SP1	sphingosine 1-phosphate
TCM	central-memory T cells (CD45RA-CCR7+CD28+CD27+)
TEM1	effector-memory T cells subset 1 (CD45RA-CCR7-CD28+CD27+)
TEM2	effector-memory T cells subset 2 (CD45RA-CCR7-CD28+CD27-)
TEM3	effector-memory T cells subset 3 (CD45RA-CCR7-CD28-CD27+)
TEM4	effector-memory T cells subset 4 (CD45RA-CCR7-CD28-CD27-)
TEMRA	effector-memory T cells re-expressing CD45RA (CD45RA+CCR7-CD28-CD27-)
TN	naive T cells (CD45RA+CCR7+CD28+CD27+)
TNFα	tumour necrosis factor α
TRM	tissue resident memory T cells
TX	transplantation
VP1	virion protein 1

Table S1. Monoclonal antibodies used for phenotyping.

anti	clone	fluorochrome	staining	manufacturer
**CD3**	UCHT1	V500	surface	BD Bioscience[1]
**CD4**	SK3	BUV563	surface	BD Bioscience[1]
**CD8**	RPA-T8	BV785	surface	Sony Biotechnology[2]
**CD27**	M-T271	APC	surface	BD Bioscience[1]
**CD28**	CD28.2	APC-R700	surface	BD Bioscience[1]
**CD45**	HI30	PerCP-eFluor 710	surface	eBioscience[3]
**CD45RA**	HI100	BV650	surface	BD Bioscience[1]
**CD69**	FN50	APC-Fire 750	surface	Biolegend[4]
**CD103**	Ber-ACT8	BUV395	surface	BD Bioscience[1]
**CD103**	Ber-ACT8	BV711	surface	BD Bioscience[1]
**CD127 (IL-7Rα)**	eBioRDR5	PE-Cy7	surface	eBioscience[3]
**CD183 (CXCR3)**	REA232	PE-Vio 615	surface	Miltenyi Biotec[5]
**CD186 (CXCR6)**	K041E5	PE-Cy7	surface	Biolegend[4]
**CD197 (CCR7)**	2-L1-A	PE-CF594	surface	BD Bioscience[1]
**KLRG1**	3F12F2	AF488	surface	eBioscience[3]
**ki-67**	Ki-67	BV650	intracellulair	BD Bioscience[1]
**granzyme B**	GB11	AF700	intracellulair	BD Bioscience[1]
**T-bet**	4B10	BV711	intracellulair	Biolegend[4]
**eomes**	WD1928	eFluor660	intracellulair	eBioscience[3]

[1]Franklin Lakes, NJ, USA [2]Weybridge, UK [3]Thermo Fisher Scientific, San Diego, CA, USA [4]San Diego, CA, USA [5]Bergisch Gladbach, Germany

Table S2. Monoclonal antibodies used for stimulation assay.

anti	clone	fluorochrome	staining	manufacturer
**CD3**	SK7	PerCP-eFluor 710	surface	eBioscience Inc.[2]
**CD4**	RPA-T4	BV711	surface	BioLegend[4]
**CD8**	RPA-T8	BV785	surface	Sony Biotechnology[3]
**CD103**	Ber-ACT8	BB515	surface	BD Bioscience1
**CD161**	HP-3G10	PE-Cy7	surface	BioLegend[4]
**GM-CSF**	BVD2-21C11	PE-Dazzle	intracellular	BioLegend[4]
**IFNγ**	B27	BUV 395	intracellular	BD Bioscience[1]
**IL-2**	MQ1-17H12	BV510	intracellular	BioLegend[4]
**IL-17A**	N49-653	BV650	intracellular	BD Bioscience[1]
**TNFα**	Mab11	AF700	intracellular	BD Bioscience[1]

[1]Franklin Lakes, NJ, USA [2]Thermo Fisher Scientific, San Diego, CA, USA [3]Weybridge, UK [4]San Diego, CA, USA

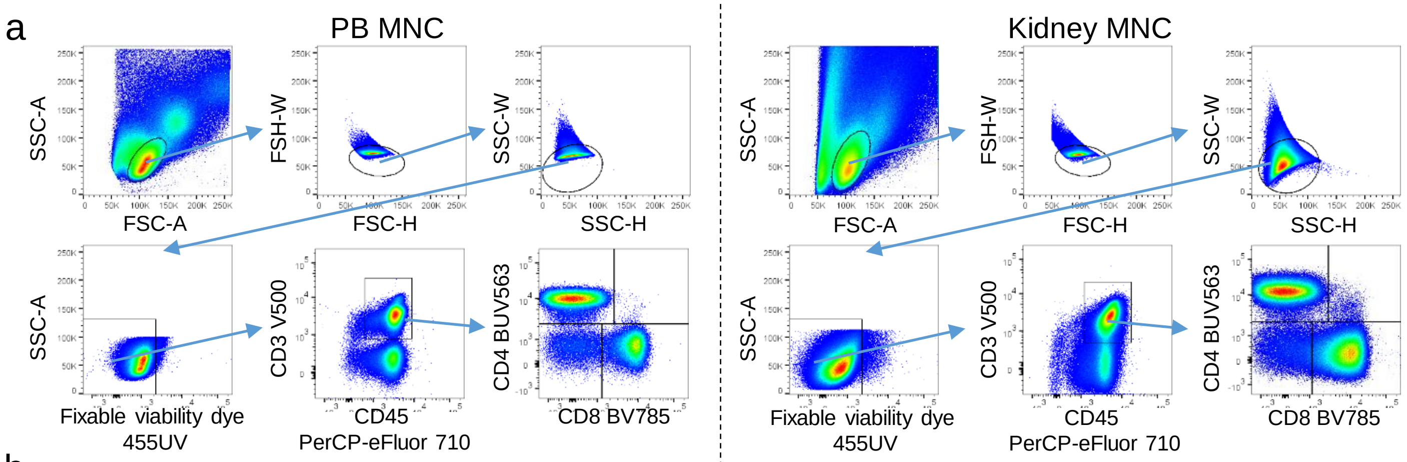

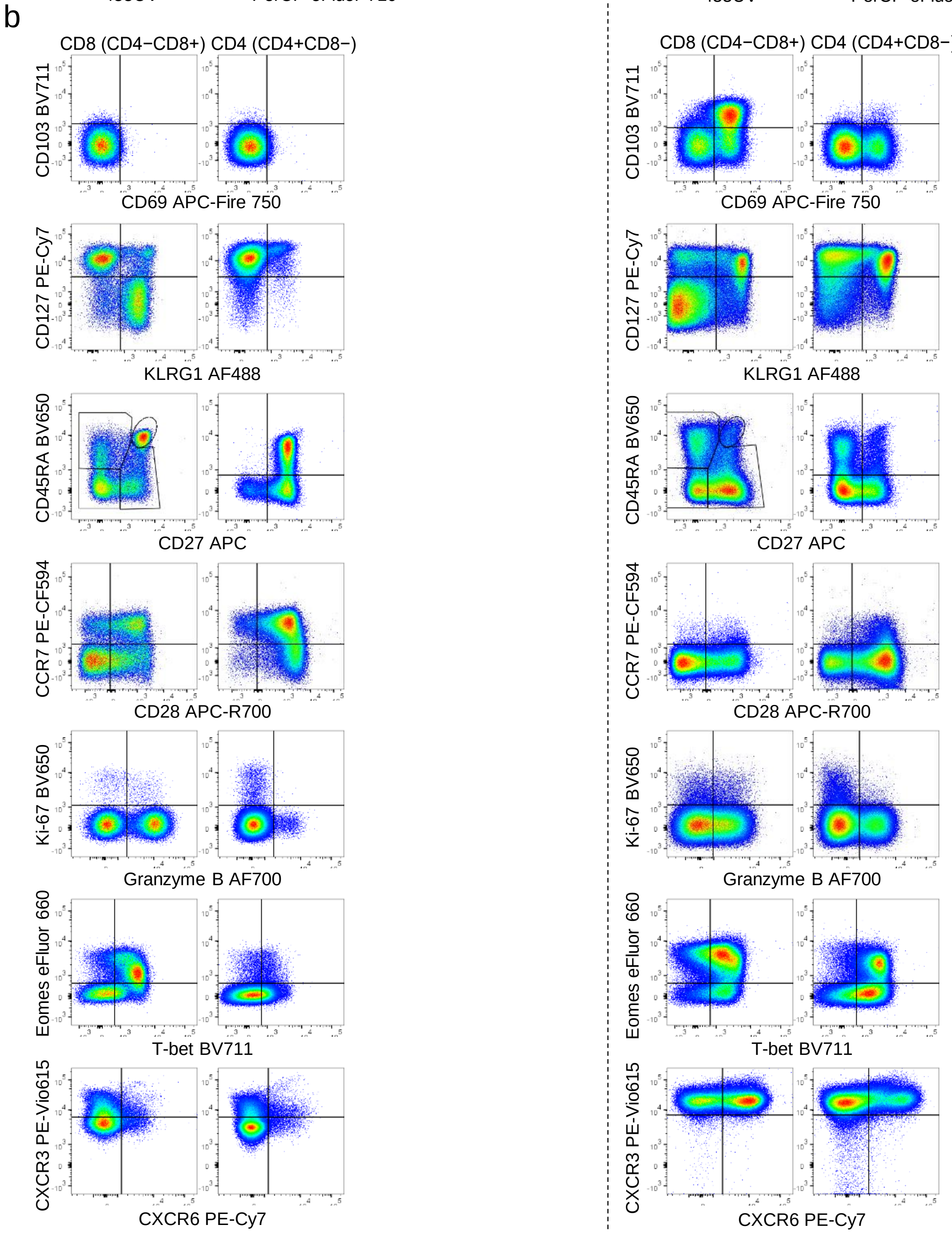

Figure S1. Gating strategy T cell phenotyping (**a**) Gatingstrategy of CD8 ($CD4^{-}CD8^{+}$) and CD4 ($CD4^{+}CD8^{-}$), T cells in live $CD45^{+}CD3^{+}$ single cell lymphocytes in PB (left) and kidney-derived MNC (right). (**b**) Gating strategy within CD8 and CD4 T cells of (from top to bottom) CD69 vs CD103, KLRG1 vs CD127 (IL-7Rα), CD27 vs CD45RA, CD28 vs CCR7, granzyme B vs Ki-67, T-bet vs eomes and CXCR6 vs CXCR3.

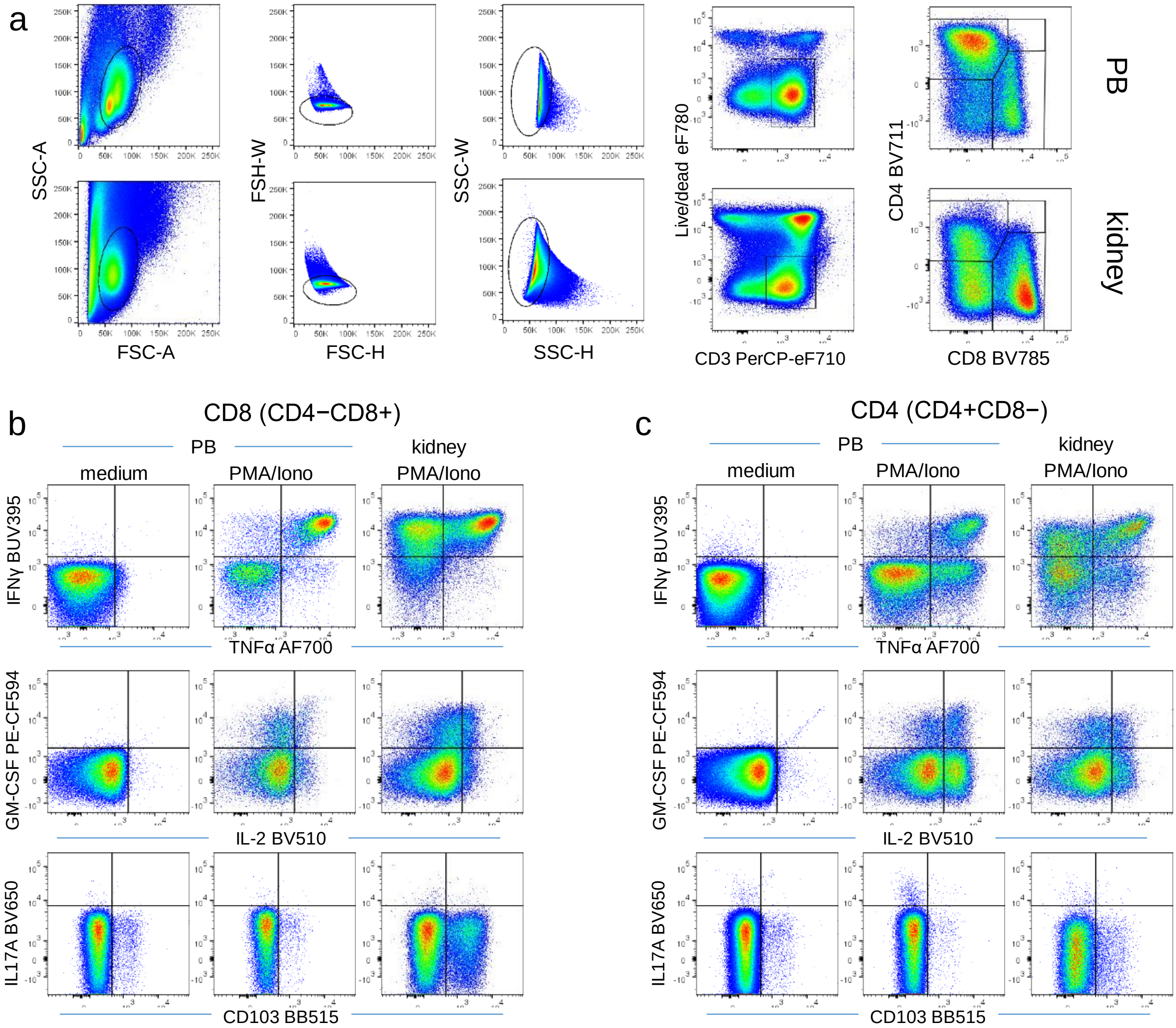

Figure S2. Gating strategy polyfunctional assay

(**a**) Gatingstrategy of CD8 ($CD4^{-}CD8^{+}$) and CD4 ($CD4^{+}CD8^{-}$) T cells in live $CD3^{+}$ single cell lymphocytes in peripheral blood (PB) (top row) and kidney-derived MNC (bottom row) after stimulation with PMA and ionomycin for 4 hrs. (**b**–**c**) Gating strategy within CD8 (**b**) and CD4 (**c**) of (from top to bottom) TNFα vs IFNγ, IL-2 vs GM-CSF and CD103 vs IL17A.

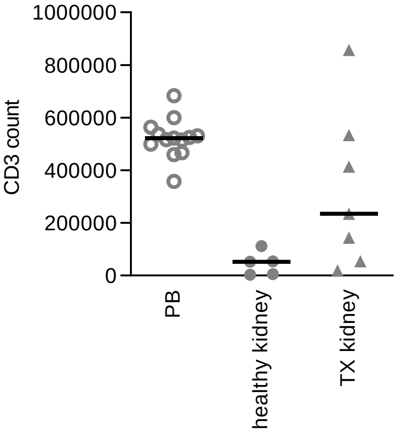

Figure S3. CD3 cell counts
Scatterplots of the CD3$^+$ cell counts analyzed per sample in healthy PBMCs, healthy kidney MNCs and TX kidney MNCs.
No statistical comparisons were made. The horizontal dash represents the median.

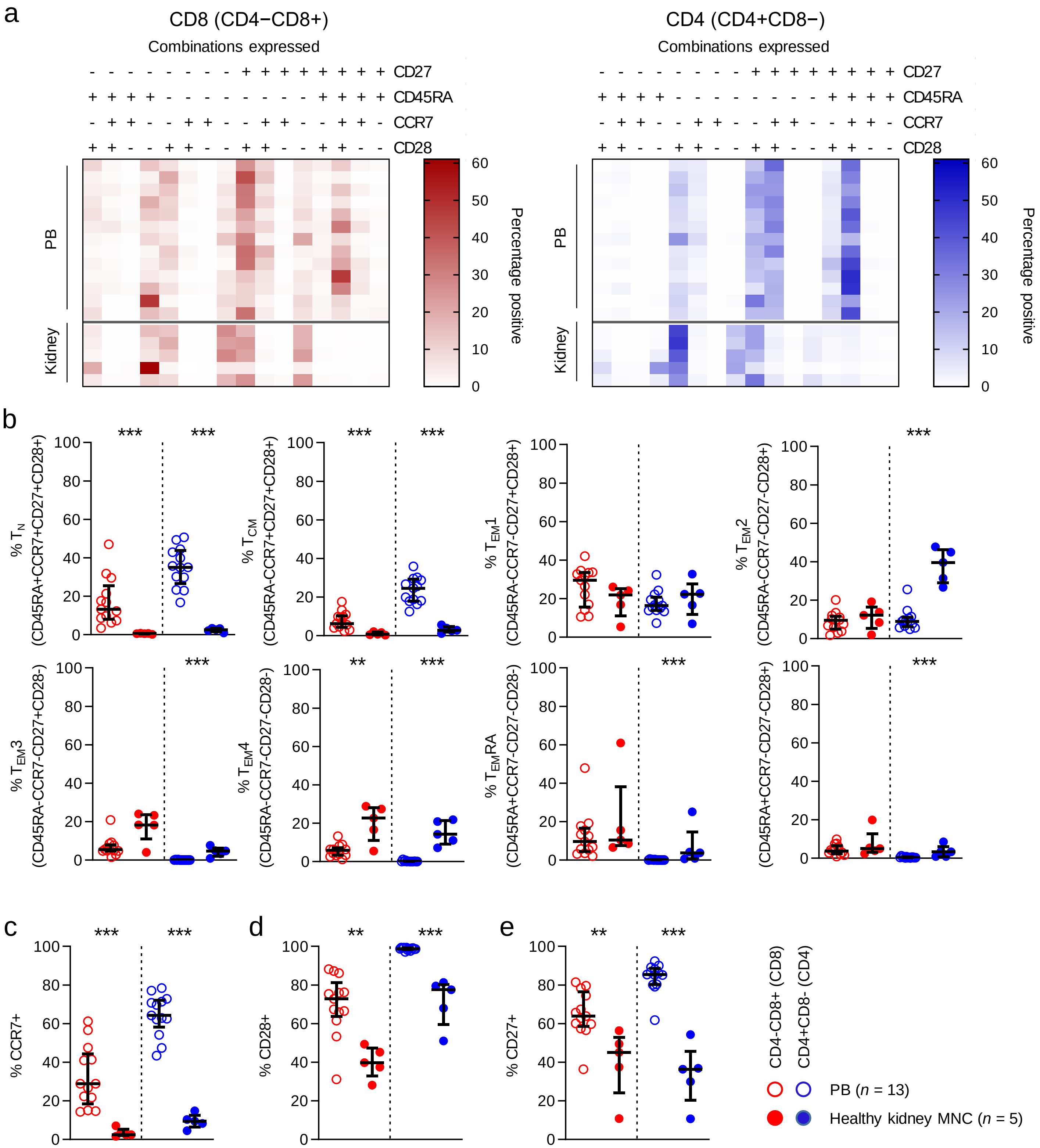

Figure S4 (supplemental data with figures 1 and 2): Phenotype of CD8 and CD4 T cells derived from PB and healthy kidney.
(**a**) Heatmap of the individual expression patterns of CD27, CD45RA, CCR7 and CD28 within CD8 and CD4 T cells from peripheral blood (PB) and healthy kidney. (**b**) Individual comparison of the 7 largest populations from heatmap a (median with IQR in black). (**c–e**) Comparison of total expression of CCR7 (**b**), CD28 (**c**) and CD27 (**d**) in CD8 (red) and CD4 (blue) T cells derived from peripheral blood (PB) and kidney (median with IQR in black). Mann-Whitney U-test was used for statistical comparison. Only significant *p*-values are displayed: **$p \leq 0.01$, ***$p \leq 0.001$.

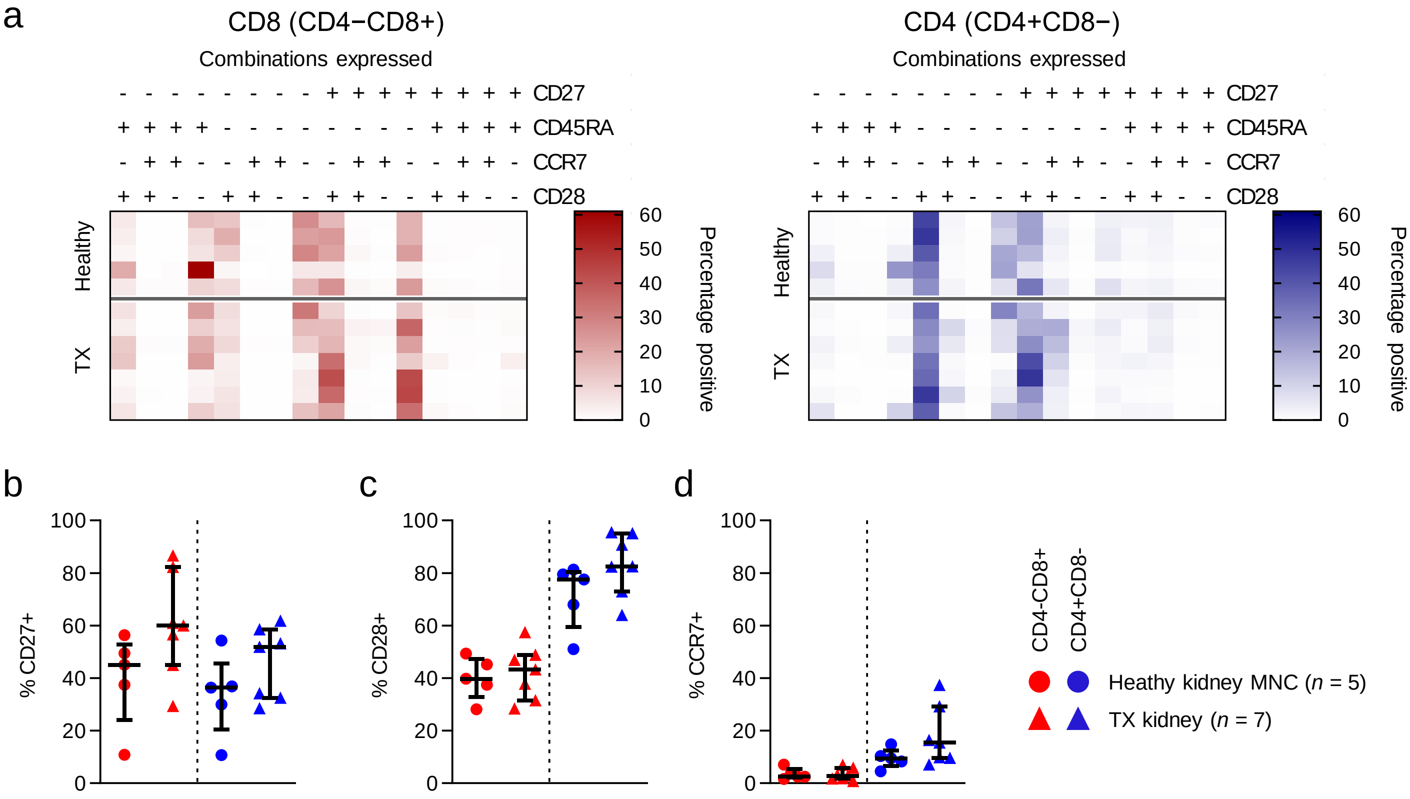

Figure S5 (supplemental data with figure 3): CD27/CD45RA/CCR7/CD28-expression profile and phenotype of CD4 and CD8 T cells from healthy kidney compared to TX kidney.
(**a**) Heatmap of the individual expression patterns of CD27, CD45RA, CCR7 and CD28 within CD4 and CD8 T cells from healthy kidney and TX (transplanted) kidney. (**b–d**) Comparison of expression of CD27 (**b**) and CD28 (**c**), CCR7 (**d**) in CD8 (red) and CD4 (blue) T cells derived from healthy kidney and TX (transplanted) kidney (median with IQR in black). Mann-Whitney U-test was used for statistical comparison. Only significant p-values are displayed: *$p < 0.05$, **$p \leq 0.01$, ***$p \leq 0.001$, ****$p \leq 0.0001$.

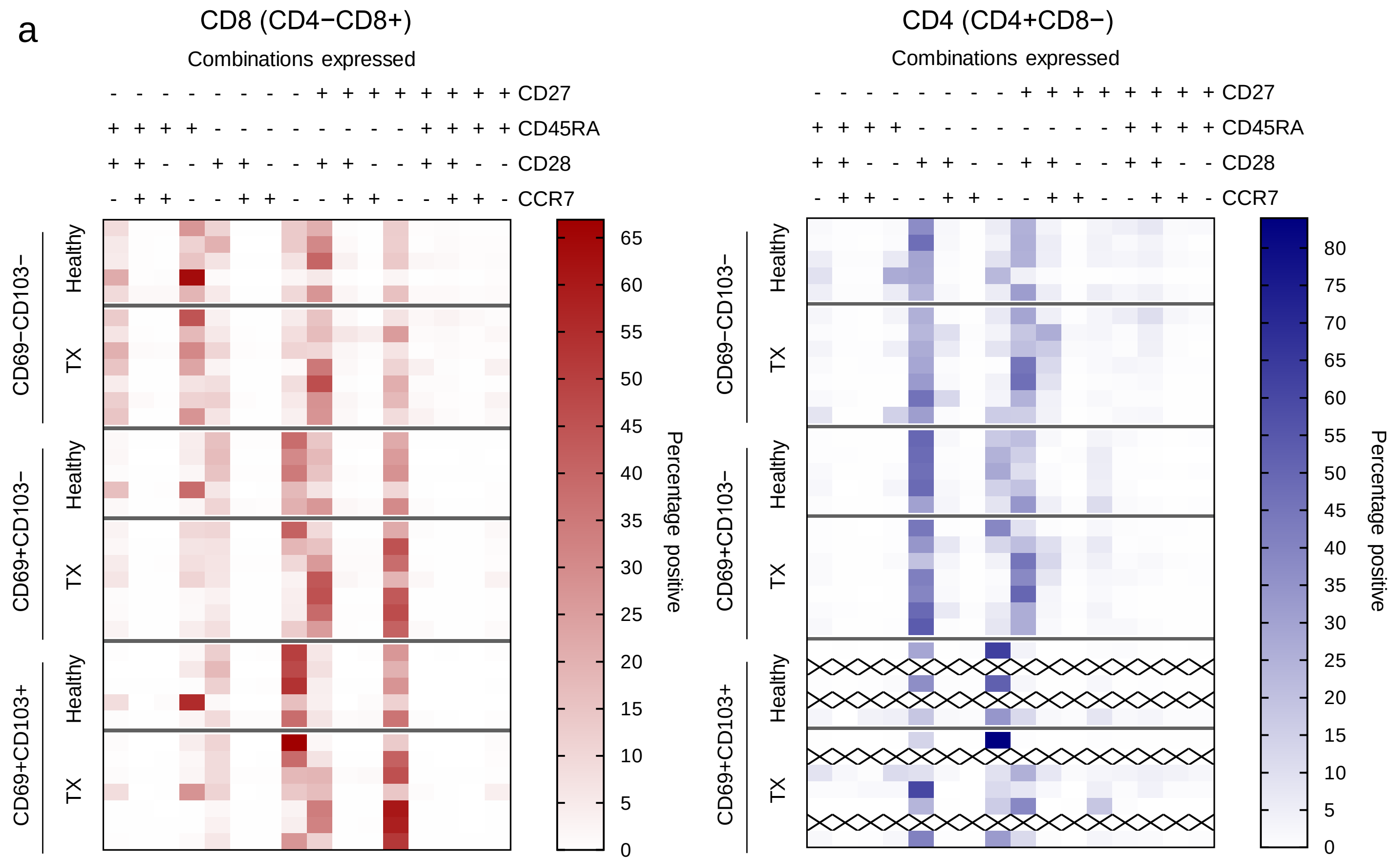

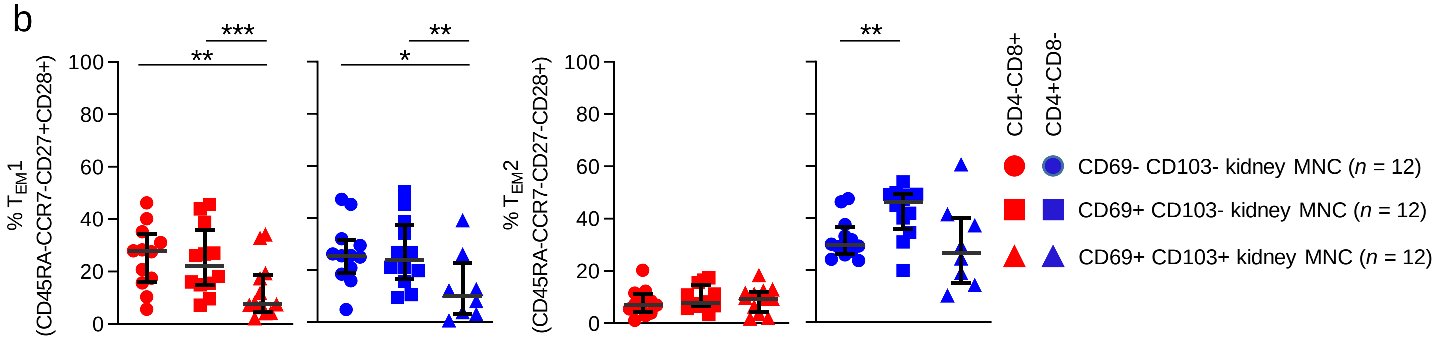

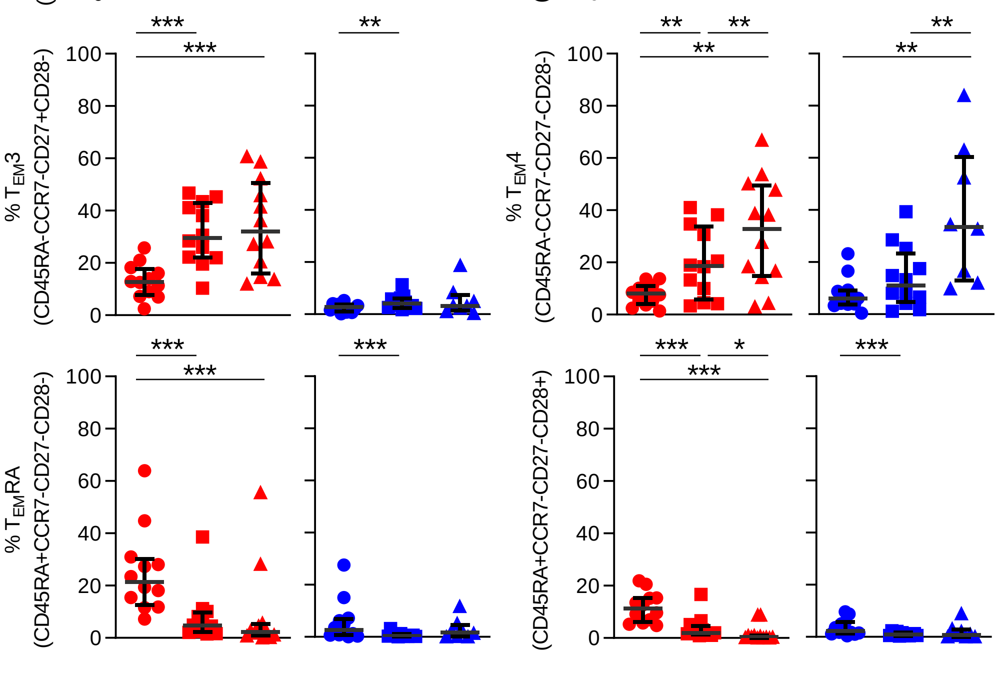

Figure S6 (supplemental data with figure 4): Phenotype of CD69/CD103 based subsets in CD8 and CD4 T cells derived from kidney.
(**a**) Heatmap of the individual expression patterns of CD27, CD45RA, CCR7 and CD28 within CD69$^-$CD103$^-$, CD69$^+$CD103$^-$ and CD69$^+$CD103$^+$ subsets within CD8 and CD4 T cells derived from (healthy and TX (transplanted)) kidney. (**b**) Individual comparison of the 5 largest populations from heatmap a (median with IQR in black). Wilcoxon signed rank test was used for statistical comparison. Only significant *p*-values are displayed: $^*p < 0.05$, $^{**}p \leq 0.01$, $^{***}p \leq 0.001$.

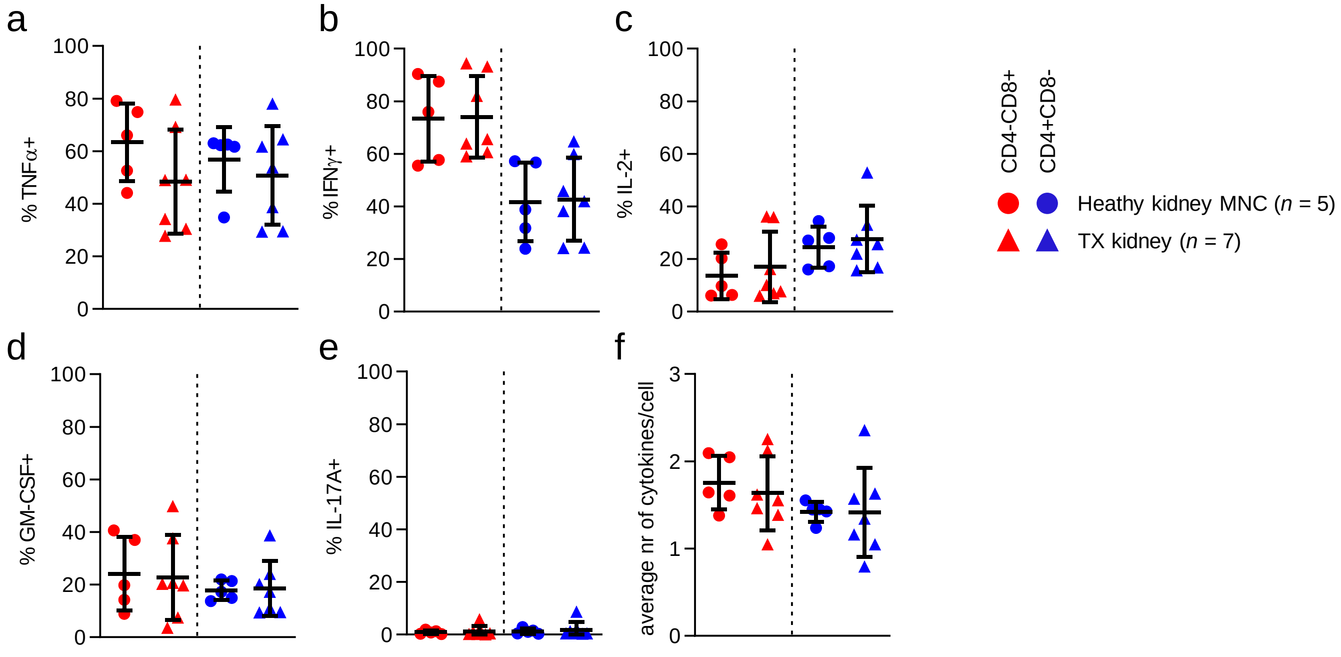

Figure S7 (supplemental data with figure 7): Cytokines produced by healthy kidney—compared to TX kidney —derived CD8 and CD4 T cells.
(**a**–**e**) Percentage of TNFα-(**a**), IFNγ-(**b**), IL-2-(**c**), GM-CSF-(**d**) and IL-17A-(**e**) producing CD8 (red) and CD4 (blue) T cells derived from healthy and transplanted (TX) kidney after 4 hr stimulation with PMA and ionomycin (median with IQR in black). (**f**) The average number of cytokines produced by CD8 and CD4 T cells derived from healthy and TX kidney (median with IQR in black). Mann-Whitney U-test was used for statistical comparison. No statistical differences were found.
